# Antihypertensive Activity of *Eucommia Ulmoides* Oliv: Male Flower Extract in Spontaneously Hypertensive Rats

**DOI:** 10.1155/2020/6432173

**Published:** 2020-04-30

**Authors:** Zhen-Jiang Ding, Chao Liang, Xiao Wang, Xin Yao, Ruo-Han Yang, Zhan-Sheng Zhang, Jin-Jin He, Hong-Yan Du, Dong Fang, Qin Li

**Affiliations:** ^1^School of Pharmaceutical Science, Henan University, Kaifeng, China; ^2^The First Affiliated Hospital of Henan University, Henan University, Kaifeng 475001, China; ^3^Paulownia Research and Development Center, State Forestry Administration, Zhengzhou, China

## Abstract

*Eucommia ulmoides* Oliv. is a traditional medical plant in Asia; however, it is still unknown whether *Eucommia* male flowers have an antihypertensive activity. In this study, we found that the aqueous extract of *Eucommia ulmoides* Oliv. male flowers can lower the blood pressure of SHR in a dose-dependent manner. Mechanistic studies suggested that the aqueous extract of male flowers can promote the mRNA and protein expressions of ACE2 in the kidney of SHR. ELISA assay showed that the plasma levels of ANG II was decreased, while ANG-(1–7) was increased in SHR treated with the aqueous extract of male flowers. ACE2 inhibitor DX600 can reverse the aqueous extract of *Eucommia ulmoides* Oliv. male flower-induced downregulation of Ang II and upregulation of Ang-(1–7), as well as the reduction of blood pressure in SHR. Moreover, Ang-(1–7)-Mas receptor antagonist A-779 abolished the antihypertensive effects of the aqueous extract of *Eucommia ulmoides* Oliv. male flower in SHR. The aqueous extract of *Eucommia ulmoides* Oliv. male flowers exhibited an antihypertensive action through the activation of ACE2-Ang-(1–7)-Mas signaling pathways in spontaneously hypertensive rats.

## 1. Introduction

Hypertension is a major risk factor that can lead to cardiac failure, coronary artery disease, stroke, renal injury, and even mortality [1, 2]. According to World Health Organization (WHO), hypertension affects more than 1 billion people over the world and accounts for about 12.8% of all annual deaths globally [3–5]. Although many effective synthesis drugs have been employed in hypertension treatment, undesirable adverse drug reactions usually appear after long-term administration. Therefore, there is a great deal of interest in using natural plant extracts as an alternative treatment for hypertension.

*Eucommia ulmoides* Oliv., also called Du-Zhong, is a traditional medical plant in China, Korea, and Japan [6, 7]. Accumulating evidences suggested *Eucommia ulmoides* Oliv. can exert several pharmacological effects such as anti-inflammatory, antioxidant, antibacterial, and anti-hyperglycemic activities [7, 8]. Especially, the cortex of *Eucommia ulmoides* Oliv. has been widely used as an appealing candidate treatment for hypertention [9]. However, the annual yield of Eucommiae Cortex is rather low, which greatly limits its application as a traditional tonic medicine against hypertension. Unlike Eucommiae Cortex, *Eucommia* male flowers are available in relatively large yields and can be harvested every year [6]. Previous researches have demonstrated that *Eucommia* male flowers provide a range of beneflts, including anti-inflammatory, analgesic, and antibacterial effects. However, to the best of our knowledge, there is no pharmacological study conducted for testing the antihypertensive ability of *Eucommia* male flowers until now.

The pathway of angiotensin-converting enzyme 2/angiotensin (1–7)/Mas (ACE2/Ang-(1–7)/Mas) in the kidney plays a critical role in blood pressure regulation [10, 11]. As a member of renin-angiotensin system (RAS), ACE2 acts as a carboxypeptidase to catalyze the conversion of ANG II into ANG-(1–7) [12, 13]. Then, the heptapeptide ANG-(1–7) binds to the G protein-coupled receptor MasR and exhibits antihypertensive activity [14]. However, it is still unclear whether *Eucommia* male flowers could affect the ACE2/Ang-(1–7)/Mas pathway in hypertension.

Thus, this study aims to investigate the antihypertensive activity of the male flower extract and explore the underlying mechanisms of antihypertension in spontaneously hypertensive rats.

## 2. Materials and Methods

### 2.1. Plant Material and Preparation of the Aqueous Extract

*Eucommia* male flowers were purchased from Lingbao City, Henan Province, China. The flowers were dried at room temperature and reduced to powder. 100 g of powdered male flowers mixed with 1 L distilled water was boiled for 1 h, and the solution was collected. Then, the residue was reboiled with 1 L distilled water for another 1 h, and the solution was collected. Finally, the solution was combined and filtered using a Millipore filter (Millipore 0.2 mm, St Quentin en Yvelines, France). The filtration of the extract was lyophilized in a freeze dryer so that 1 ml of the extract corresponded to 0.2 g of dried flowers. Before administration to rats, the filtration of the extract was diluted into desired concentrations with distilled water.

### 2.2. Experimental Animals

Male spontaneously hypertensive rats (8-week-old) and Sprague Dawley (SD) rats (8 weeks old) were obtained from Beijing Weitong Lihua Animal Co. All rats were housed three per cage with free access to food and water in a room kept at 20°C–24°C with 12 h light/dark cycles. Rats were acclimatized to the environment before experiment. All animal procedures were performed in accordance with the guidelines of the Institutional Animal Care and Use Committee of Henan University.

### 2.3. Drug Administration and Blood Measurement

To evaluate the antihypertensive effects of the aqueous extract of *Eucommia ulmoides* Oliv. male flowers, male spontaneously hypertensive rats were randomly divided into the following four groups: hypertensive group (distilled water), high-dose group (0.20 g/mL flower extract), medium-dose group (0.10 g/mL flower extract), and low-dose group (0.05 g/mL flower extract); the drugs or water was administrated orally at a dose of 1.0 mL/kg once daily for 7 weeks. The doses of drugs were determined based on our preliminary experiments since there are few studies on the male flowers of *Eucommia ulmoides*. Normal male Sprague Dawley rats were used as a normal control. Systolic blood pressure was measured just before the aqueous extract administration and then was measured once a week for 7 weeks after administration by the noninvasive tail-cuff method using a blood pressure monitor (Shanghai Alcott Biotech Co., Ltd., Shanghai, China). Before the test of blood pressure, the rats were kept at around 30°C for 15 min. At least five measurements of blood pressure were taken and were averaged as the result of each rat. In order to minimize stress-induced variations in blood pressure, all measurements were taken by the same individual at the same time under the same conditions.

To determine whether the ACE2/Ang-(1–7)/Mas pathway was involved in the antihypertensive effects of the aqueous extract of male flowers, ACE2 inhibitor DX600 (5 *μ*g/kg/day) or Ang-(1–7)/Mas inhibitor A779 (1 mg/kg/day) was administrated intraperitoneally to rats 30 minutes before the application of aqueous extract of male flower (0.20 g/mL flower extract) once per day for 7 weeks. The doses of ACE2 inhibitor DX600 and Ang-(1–7)/Mas inhibitor A779 were determined according to the previous report [15]. Then, the systolic blood pressure was measured by the noninvasive tail-cuff method using a blood pressure monitor as described above.

### 2.4. Measurements of Plasma Ang II and Ang-(1–7)

Plasma Ang II and Ang-(1–7) were measured with the rat enzyme-linked immunosorbent assay (ELISA) kits (Nanjing Jiancheng Bioengineering Institute, China) according to the instructions of the manufacturer.

### 2.5. Real-Time PCR

RNA from the left kidney was extracted using the TRIzol reagent (Invitrogen, Carlsbad, CA, USA). Complementary DNA was synthesized by using random hexamers and MMLV reverse transcriptase according to the manufacturer's instructions (Takara, Tokyo, Japan). Relative quantitative real-time PCR was performed using 2 × SYBR Green PCR master Mix (Promega) on an ABI 7500 sequence detection system (Applied Biosystems). The reaction conditions were as follows: 95°C, pre-denaturation for 5 min, 15 seconds at 95°C, and 1 minute at 60°C for a total of 40 cycles. Glyceraldehyde phosphate dehydrogenase (GAPDH) was used as an internal control. The relative expression level of the genes was calculated by the 2^−ΔΔCt^ method. Specific primers for the rat were as follows: ACE2, 5′-CGC TGT CAC CAG ACA AGAA-3′ (forward) and 5′-CGT CCA ATC CTG GTT CAAG-3′ (reverse); GAPDH, 5′- AGC CAT GTA CGT AGC CAT CC -3′(forward), and 5′- GCC ATC TCT TGC TCG AAG TC -3′(reverse).

### 2.6. Western Blot

Western blot analysis was conducted as previously described [16]. Briefly, the total proteins were extracted from left ventricles using ice-chilled RIPA lysis buffer containing 50 mM Tris-HCl (pH 8.0), 150 mM NaCl, 0.5% sodium deoxycholate, 0.1% SDS, 1% NP-40, 5 mM EDTA, 0.25 mM PMSF, and protease inhibitor cocktail. The concentration of total protein was determined using a BCA assay kit (Pierce, Rockford, IL). Protein samples were denatured and separated through SDS-polyacrylamide gel electrophoresis (SDS-PAGE). Following separation, the protein was transferred onto a PVDF membrane. The membranes were then blocked with 5% nonfat in Tris-buffered saline and Tween (TBST) (20 mM Tris-HCl, pH 7.5, 150 mM NaCl, and 0.05% Tween-20) for 1 h at room temperature. After blocking, the membrane was incubated with rabbit monoclonal anti-ACE2 (1 : 1000; ab108252; Abcam, Cambridge, UK) and mouse monoclonal anti-*β*-actin antibody (1 : 2000; sc-47778; Santa Cruz, CA, USA) at 4°C overnight. The blots were washed in TBST and then were incubated in horseradish peroxidase-conjugated goat anti-rabbit (1 : 1000, sc-2004)/mouse IgG secondary antibody (1 : 1000, sc-2005) for 1 h at room temperature. Finally, the blots were detected using the ECL plus reagents and visualized using a FluroChem E Imager (Protein Simple, San Jose, CA, USA).

### 2.7. Statistical Analysis

Statistical analysis was performed using the GraphPad Prism 5.0 (GraphPad Software, Inc., La Jolla, CA, USA). All data were expressed as mean ± SEM. One-way analysis of variance (ANOVA) followed by Tukey or Dunnet's posttests or two-way ANOVA followed by the Bonferroni post hoc test was used to compare means of multiple experimental groups. A two-tailed unpaired *t* test was used for the comparison of the mean values between two groups. *P* < 0.05 was considered to be significant.

## 3. Results

### 3.1. Effects of the Aqueous Extract of *Eucommia Ulmoides* Oliv. on Blood Pressure in SHR Rats

**Figure 1:**
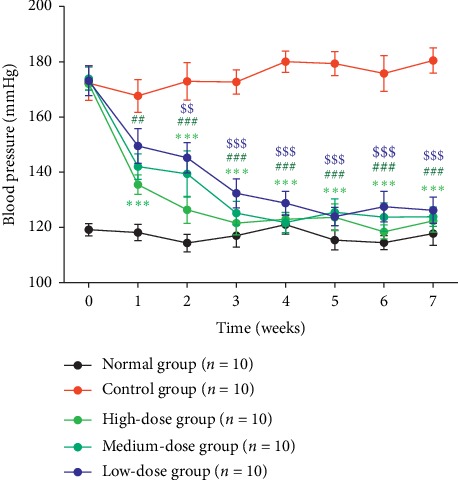
Effects of 7-week treatment with *Eucommia ulmoides* Oliv. male flower extract on systolic blood pressure in SHRs. Data were expressed as the mean ± SEM. ^*∗∗∗*^*P* < 0.001 (high-dose group) compared with the control group; ^##^*P* < 0.01, ^###^*P* < 0.001 (medium-dose group) compared with the control group; ^$$^*P* < 0.01, ^$$$^*P* < 0.001 (low-dose group) compared with the control group; 2-way analysis of variance.

To investigate whether the aqueous extract of *Eucommia ulmoides* Oliv. male flowers exhibit antihypertensive activity in spontaneously hypertensive rats, we administrated the aqueous extract of *Eucommia ulmoides* Oliv. male flowers to spontaneously hypertensive rats and measured its effect on blood pressure. As shown in Figure 1, after treated with the aqueous extract of *Eucommia ulmoides* Oliv., the blood pressure of SHR rats was reduced in a dose-dependent manner. For example, before the aqueous extract administration, the values of blood pressure were nearly equal among the low-dose group (173.14 ± 5.40 mmHg), medium-dose group (173.86 ± 4.16 mmHg), and high-dose group (172.04 ± 2.28 mmHg). After 1 week administration, the blood pressure of SHR rats was decreased to 149.54 ± 6.28 mmHg at low dose, 142.04 ± 4.60 mmHg at medium dose, and 135.50 ± 3.56 mmHg at high dose; After 2 weeks administration, the blood pressure was decreased to 145.25 ± 5.43 mmHg at low dose, 139.39 ± 8.36 mmHg at medium dose, and 126.38 ± 4.88 mmHg at high dose; after 3 weeks administration, the blood pressure was decreased to 132.40 ± 5.24 mmHg at low dose, 125.19 ± 4.29 mmHg at medium dose, and 121.60 ± 4.29 mmHg at high dose. Then, after 4 weeks to 7 weeks' administration, the values of blood pressure were nearly reduced to the same degree among the three groups.

### 3.2. Aqueous Extract of *Eucommia Ulmoides* Oliv. Male Flower Exhibits Antihypertensive Effects through Promoting ACE2 Expression in Spontaneously Hypertensive Rats

**Figure 2:**
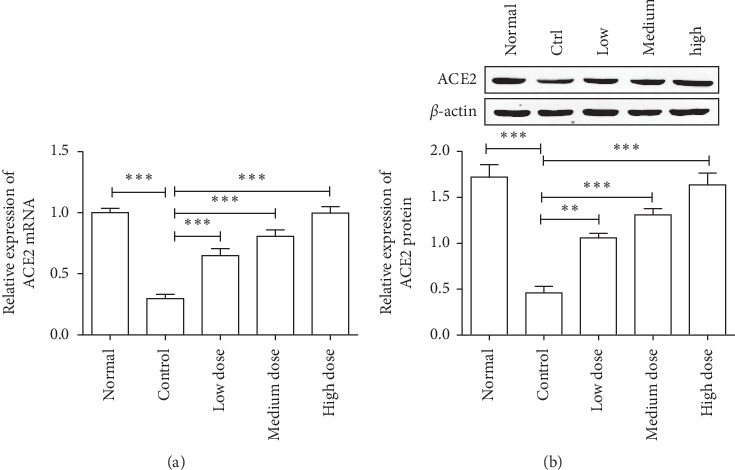
*Eucommia ulmoides* Oliv. male flower extract can promote the expression of ACE2 mRNA (a) and protein (b) in SHR. ^*∗∗*^*P* < 0.01, ^*∗∗∗*^*P* < 0.001, 1-way analysis of variance, *n* = 8/group.

**Figure 3:**
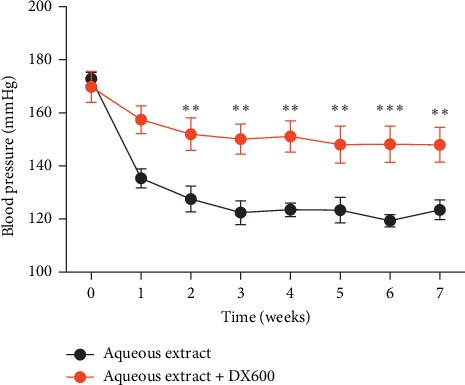
ACE2 inhibitor DX600 can reverse the blood pressure reduction induced by the aqueous extract of *Eucommia ulmoides* Oliv. male flower in SHR. ^*∗∗*^*P* < 0.01,^*∗∗∗*^*P* < 0.001, 2-way analysis of variance, *n* = 8/group.

To explore the antihypertensive mechanisms of *Eucommia ulmoides* Oliv. male flower extract, we first examined the expression of ACE2 in the kidney in spontaneously hypertensive rats after treating with the aqueous extract of *Eucommia ulmoides* Oliv. male flowers. As shown in Figure 2, the mRNA and protein expressions of ACE2 were downregulated in spontaneously hypertensive rats compared with normal rats; while the aqueous extract of Eucommia ulmoides Oliv. male flower can promote the mRNA and protein expression of ACE2 in spontaneously hypertensive rats. Moreover, we also found that administration of ACE2 inhibitor DX600 can reverse the blood pressure reduction induced by the aqueous extract of *Eucommia ulmoides* Oliv. male flower in spontaneously hypertensive rats (Figure 3). Obviously, these results indicated that the aqueous extract of *Eucommia ulmoides* Oliv. male flowers exhibits antihypertensive effects through promoting the ACE2 expression in SHR.

### 3.3. ACE2-Ang-(1–7)-Mas Signaling Pathways Are Involved in the Antihypertensive Effects of the Aqueous Extract of *Eucommia Ulmoides* Oliv. Male Flowers

**Figure 4:**
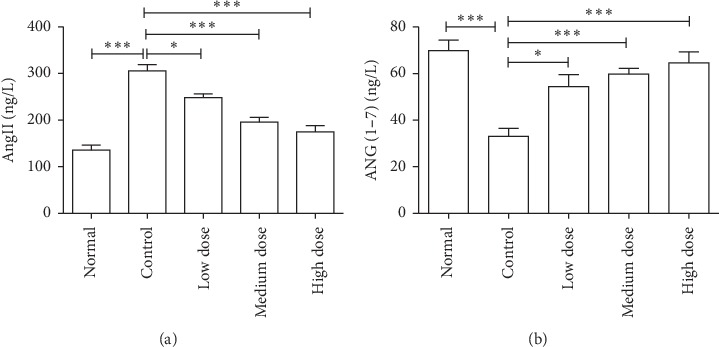
Effects of *Eucommia ulmoides* Oliv. male flowers extract of on the plasma level of Ang II (a) and Ang-(1–7) (b). Note that the extracts can reduce the level of Ang II and increase the level of Ang-(1–7). ^*∗*^*P* < 0.05, ^*∗∗∗*^*P* < 0.001, 1-way analysis of variance, *n* = 8/group.

**Figure 5:**
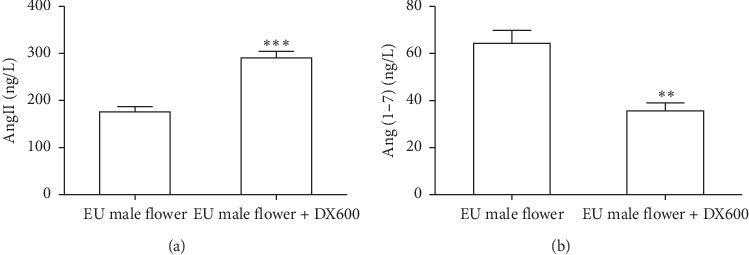
ACE2 inhibitor DX600 can reverse the aqueous extract of *Eucommia ulmoides* Oliv. male flower-induced downregulation of Ang II (a) and upregulation of Ang-(1–7) (b) in SHR. ^*∗∗*^*P* < 0.01, ^*∗∗∗*^*P* < 0.001, 2-tailed unpaired *t* test, *n* = 8/group.

ACE2 was reported to catalyze the degradation of Ang II into Ang-(1–7), which subsequently binds to Mas receptor and reduces blood pressure [17]. Considering that *Eucommia ulmoides* Oliv. male flowers can promote the expression of ACE2 in spontaneously hypertensive rats, we firstly measured the content of Ang II and Ang-(1–7) in spontaneously hypertensive rats after treating with the aqueous extract of *Eucommia ulmoides* Oliv. male flowers. As shown in Figure 4, the aqueous extract of *Eucommia ulmoides* Oliv. male flowers can decrease the level of Ang II in spontaneously hypertensive rats. By contrast, Ang-(1–7) was significantly increased in the plasma in spontaneously hypertensive rats after the administration of the aqueous extract of *Eucommia ulmoides* Oliv. male flower. Moreover, the ACE2 inhibitor DX600 can reverse the aqueous extract of *Eucommia ulmoides* Oliv. male flower-induced downregulation of Ang II and upregulation of Ang-(1–7) in spontaneously hypertensive rats (Figure 5). Collectively, these results indicated that ACE2 was involved in the aqueous extract of *Eucommia ulmoides* Oliv. male flower-induced production of Ang-(1–7) in spontaneously hypertensive rats.

**Figure 6:**
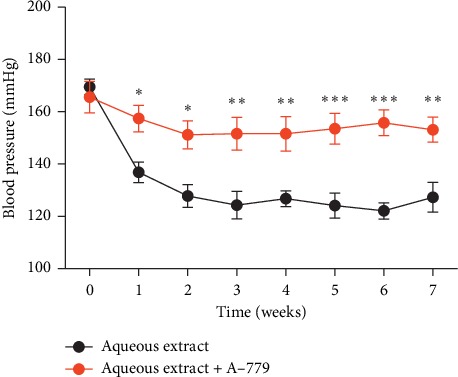
Ang-(1–7)-Mas receptor antagonist A-779 can abolish the antihypertensive effects of *Eucommia ulmoides* Oliv. male flower extract in SHR. ^*∗*^*P* < 0.05, ^*∗∗*^*P* < 0.01, ^*∗∗∗*^*P* < 0.001, 2-way analysis of variance, *n* = 8/group.

In order to identify that Ang-(1–7) contributed to the antihypertensive effects of the aqueous extract of *Eucommia ulmoides* Oliv. male flower in spontaneously hypertensive rats, we administrate the Ang-(1–7)-Mas receptor antagonist A-779 to spontaneously hypertensive rats 30 min before the aqueous extract of *Eucommia ulmoides* Oliv. male flower application and examined the blood pressure. As shown in Figure 6, the Ang-(1–7)-Mas receptor antagonist A-779 almost abolished the antihypertensive effects of the aqueous extract of *Eucommia ulmoides* Oliv. male flower in spontaneously hypertensive rats.

## 4. Discussion

*Eucommia ulmoides* Oliv. is a traditional medicinal plant which mainly spreads in Asia [18, 19]. A large number of studies have suggested that the leaf and bark of *Eucommia ulmoides* Oliv. exhibit several pharmacological effects, such as lowering blood hypertension, strengthening tendons and bones, benefiting liver and kidney, and increasing longevity [20, 21]. However, the pharmacological properties of *Eucommia* male flowers are still largely unknown. As far as we know, our results first revealed that the aqueous extracts from male flowers have an antihypertensive effect.

ACE2 is a human ACE-related carboxypeptidase which catalyzes the degradation of Ang II into a heptapeptide, Ang-(1–7) [12]. Numerous studies have reported that ACE2 could act as a suppressor in the progression of blood pressure [22, 23]. For instance, the levels of ACE2 mRNA and protein were markedly decreased in the kidneys of hypertensive rats [13, 24]. Overexpression of ACE2 in blood vessels reduced the blood pressure [25]. In this study, our findings suggest that the aqueous extract of male flowers can induce the mRNA and protein expression of ACE2 in the kidney of hypertensive rats, which in turn reduces the blood pressure. A growing number of in vivo and in vitro studies have demonstrated that Ang-(1–7) counteracts the effects of Ang II and dilates blood vessels to lower blood pressure [14, 26]. In the present study, our finding suggested that the administration of the aqueous extract of male flower promoted the degradation of Ang II into Ang-(1–7) in spontaneously hypertensive rats. However, we did not identify the antihypertensive components in the aqueous extract of male flower. Recently, many studies demonstrated that four categories extracted from the leaf and bark of EU including lignans, iridoids, flavonoids, and terpenoids have antihypertensive action [7, 27, 28], and Bessa et al. have separated iridoids and flavonoids from the male flowers of EU [12]; therefore, it is likely that iridoids and flavonoids may account for the antihypertensive effect of the male flowers of EU. Of course, further study will be needed to determine the antihypertensive components of the male flowers of EU.

In conclusion, the aqueous extract of *Eucommia ulmoides* Oliv. male flowers exhibited an antihypertensive action through the activation of ACE2-Ang-(1–7)-Mas signaling pathways in spontaneously hypertensive rats.

## Data Availability

All data generated or analysed during this study are included in this published article.
